# Bilateral Basal Ganglion Hemorrhage after Severe Olanzapine Intoxication

**DOI:** 10.1155/2020/2398721

**Published:** 2020-08-31

**Authors:** Kerstin Schweyer, Bastian Fatke, Kornelia Kreiser, Christian Rabe, Christian Seifert, Benno Ikenberg

**Affiliations:** ^1^Technical University of Munich, School of Medicine, Klinikum rechts der Isar, Department of Neurology, München, Germany; ^2^Technical University of Munich, School of Medicine, Klinikum rechts der Isar, Department of Psychiatry and Psychotherapy, München, Germany; ^3^Technical University of Munich, School of Medicine, Klinikum rechts der Isar, Department of Neuroradiology, München, Germany; ^4^Technical University of Munich, School of Medicine, Department of Clinical Toxicology & Poison Control Centre Munich, Klinikum rechts der Isar, München, Germany

## Abstract

Olanzapine is a second-generation antipsychotic drug which is generally considered safe with well therapeutic antipsychotic effects. We describe a patient suffering from bilateral intracerebral hemorrhage after severe olanzapine intoxication without underlying thrombocytopenia, arterial hypertension, or vascular malformation as cause of intracerebral hemorrhage. This raises the possibility of a direct side effect of high-dose olanzapine intake. So far, intracranial hemorrhage after olanzapine intoxication in such constellation has not been reported before. Given the high number of its prescription rates, our finding of intracranial hemorrhage after olanzapine intoxication is of high clinical relevance.

## 1. Introduction

Olanzapine is a second-generation antipsychotic drug which is widely used in the treatment of schizophrenia, bipolar disorders, and delirium [1]. Olanzapine is well tolerated and safe in therapeutic doses but could cause metabolic changes frequently [2]. Symptoms after intoxication correspond mainly to its adverse effects resulting from its pharmacological mechanism of action (affinity in particular for dopamine (D1-D4) and serotonin (5-HT_1-3_) receptors) including extrapyramidal and anticholinergic effects [3, 4].

## 2. Case Presentation

We report the case of a 51-year-old woman with a history of major depression after a suspected suicide attempt with olanzapine intoxication (maximum dose 160 × 7.5 mg of olanzapine; corresponding to 1200 mg). The legal representative of the patient signed informed consent for publication.

**Figure 1:**
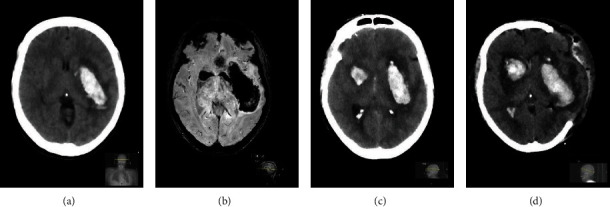
(a) Day 0. Initial CT scan presenting left-hemispheric intracranial hemorrhage in typical localization. (b) Day 2. MRI scan (SWI sequence) illustrating bilateral ICH corresponding to the CT scan. No microbleeds were detected. (c) Day 4. Progressive bilateral intracranial hemorrhage. (d). Day 4. CT scan after hemicraniectomy.

At first presentation in a primary care center, the patient was comatose and intubated. Activated charcoal was administered at an early point of time; however, no subsequent waking reaction occurred. An initial CT scan of the brain (Figure 1(a)) revealed a left-hemispheric deep intracerebral hemorrhage (ICH). Upon admission, blood pressure was in normal range with no documented history of arterial hypertension. There was no medication history of anticoagulants nor was there a reported history of alcohol or drug abuse or any preexisting diseases such as arterial hypertension or liver insufficiency. Laboratory results showed a normal platelet cell count (218/*μ*l), standard coagulation parameters (PTT: 26 s, INR: 0.9), and liver function (GPT: 30 U/l). Factor XIII was normal (85%). There were no signs of any preceding head trauma. Prior medication was olanzapine 5 mg daily and duloxetine 90 mg daily as well as lorazepam in an unknown daily dose.

For further therapy, the patient was transferred to our tertiary care center 12 hours later. At admission, we conducted a follow-up CT scan, which presented not only significant secondary enlargement of the left-hemispheric hemorrhage but also newly developed right-hemispheric ICH in the basal ganglia areas. In a CT-angiographic study, no source of the hemorrhage was detected. MRI at day 2 after admission confirmed bilateral intracerebral hemorrhage but neither showed any underlying cause of ICH (Figure 1(b)). Olanzapine serum levels measured approximately 24 hours after suspected intake were highly elevated (820 *μ*g/l) confirming severe intoxication [5]. Corrected QT interval was 450 ms.

Two days after admission, the patient's conditions improved significantly. She regained consciousness, was able to speak, and did not show any severe focal deficits. However, within a few hours, the patient again lost her consciousness, a tetraparesis occurred, and reintubation was required. Another follow-up cerebral CT scan revealed massive progression of the bilateral hemorrhage and enlargement of space-occupying edema of the left hemisphere (Figure 1(c)). There was no documented hypertensive crisis in this period. Consequently, a left-hemispheric hemicraniectomy was conducted (Figure 1(d)). In the following days, hyperpyrexia, elevation of the creatine kinase (3885 U/l), moderate leukocytosis (13.5 g/l), and muscle rigidity occurred suggesting late-onset neuroleptic malignant syndrome. In spite of medication with dantrolen, fever was lowered insufficiently. At discharge to a rehabilitation center, lastly, olanzapine intoxication was discussed as the cause of bilateral ICH, since other causes were excluded.

During a follow-up time of two months, the patient remained in a persistent vegetative state.

## 3. Discussion

Hereby, we describe the case of severe bilateral deep ICH possibly related to an olanzapine intoxication.

Simultaneous bilateral ICH is a rare condition, and such cases were related mainly to severe arterial hypertension and more rarely to trauma, tendency for bleeding, or bilateral ischemic stroke [6]. The overall prevalence of ICH is 24.6 per 100,000 person-years and increases with age [7], whereas multiple ICH were found to occur in only 2-3% percent of cases with ICH [6]. General risk factors for ICH include arterial hypertension, hematological diseases, and alcohol abuse. After excluding these conditions in our case, we attributed ICH to the preceding intoxication with olanzapine. Extended literature research on overdose effects of olanzapine showed two cases with ICH in the context of olanzapine intake. Different to our patient, in one case, ICH was associated with thrombocytopenia [8]. In another case, ICH occurred in a 38-year-old patient with hyperCKemia after suicidal olanzapine intoxication [4]. However, detailed information was not published.

Underlying mechanisms remain unclear, as so far, no directly associated vascular impact of olanzapine has been reported [9]. Considering bilateralism and secondary progression of the ICH, a systemic effect seems possible. Since laboratory results did not disclose any cause, a vascular pathology caused by overdose of olanzapine may be discussed. Secondary ICH may be explained by known prolonged toxicity of olanzapine overdose due to its lipophilic, highly protein-bound accumulation [10].

To sum up, this is the first case of ICH after olanzapine intoxication without any clear comorbidity such as hypertension or thrombocytopenia. Though no causal relation can be proven, ICH should be considered a differential diagnosis for prolonged loss of consciousness after olanzapine intoxication.

## Data Availability

Data are available from the corresponding author upon reasonable request.
